# Bone age assessment with various machine learning techniques: A systematic literature review and meta-analysis

**DOI:** 10.1371/journal.pone.0220242

**Published:** 2019-07-25

**Authors:** Ana Luiza Dallora, Peter Anderberg, Ola Kvist, Emilia Mendes, Sandra Diaz Ruiz, Johan Sanmartin Berglund

**Affiliations:** 1 Department of Health, Blekinge Institute of Technology, Karlskrona, Sweden; 2 Department of Pediatric Radiology, Karolinska University Hospital, Stockholm, Sweden; 3 Department of Computer Science, Blekinge Institute of Technology, Karlskrona, Sweden; University of Craiova, ROMANIA

## Abstract

**BACKGROUND:**

The assessment of bone age and skeletal maturity and its comparison to chronological age is an important task in the medical environment for the diagnosis of pediatric endocrinology, orthodontics and orthopedic disorders, and legal environment in what concerns if an individual is a minor or not when there is a lack of documents. Being a time-consuming activity that can be prone to inter- and intra-rater variability, the use of methods which can automate it, like Machine Learning techniques, is of value.

**OBJECTIVE:**

The goal of this paper is to present the state of the art evidence, trends and gaps in the research related to bone age assessment studies that make use of Machine Learning techniques.

**METHOD:**

A systematic literature review was carried out, starting with the writing of the protocol, followed by searches on three databases: Pubmed, Scopus and Web of Science to identify the relevant evidence related to bone age assessment using Machine Learning techniques. One round of backward snowballing was performed to find additional studies. A quality assessment was performed on the selected studies to check for bias and low quality studies, which were removed. Data was extracted from the included studies to build summary tables. Lastly, a meta-analysis was performed on the performances of the selected studies.

**RESULTS:**

26 studies constituted the final set of included studies. Most of them proposed automatic systems for bone age assessment and investigated methods for bone age assessment based on hand and wrist radiographs. The samples used in the studies were mostly comprehensive or bordered the age of 18, and the data origin was in most of cases from United States and West Europe. Few studies explored ethnic differences.

**CONCLUSIONS:**

There is a clear focus of the research on bone age assessment methods based on radiographs whilst other types of medical imaging without radiation exposure (e.g. magnetic resonance imaging) are not much explored in the literature. Also, socioeconomic and other aspects that could influence in bone age were not addressed in the literature. Finally, studies that make use of more than one region of interest for bone age assessment are scarce.

## Introduction

### Motivation

Bone development is the process that drives changes in bones’ size, shape and degree of mineralization. This happens in primary and secondary ossification centers, namely diaphysis and epiphysis, respectively, where cartilage gradually turns into bone tissue. This process persists as long as cartilage remains in the growth plate (or epiphyseal plate). At the end of the bone development process the epiphyseal plate is ossified, indicating that the diaphysis and epiphysis are fused [1].

Other important concepts that relate to such topic are skeletal maturity, bone age and chronological age. Skeletal maturity refers to the stage of development in which the bone is currently in [1]. Bone age is a closely related concept to skeletal maturity, and relates to the estimation of an age based on an individual’s skeletal maturity [2], whereas the chronological age is calculated based solely on an individual’s date of birth.

The importance of assessing an individual’s skeletal maturity or bone age and its comparison with their chronological age arises from two main angles: First, from a medical angle, the assessment of bone age is useful for the diagnosis and treatment of pediatric endocrinology, orthodontics and pediatric orthopedic disorders, in addition to also being considered in estimations of an individual’s final height [3]. Second, from a legal standpoint, bone age assessment is important in order to identify whether an individual is a minor in a situation where verified documents are lacking.

This latter aspect is extremely important given the growth in number of asylum seekers in Europe, where unaccompanied individuals under the age of 18 are granted special rights by the United Nations (UN) Convention on the Rights of the Child [4], which relate to reception arrangements, access to health care, education etc [5].

A report from the United Nations Refugee Agency (UNHCR) stated that individuals under the age of 18 contributed to 52% of the refugees population in 2017; and also in 2017 around 173,800 refugees children were unaccompanied or separated from their parents, in what the UN considers a “conservative estimate” [6]. Given such circumstances, the assessment of bone age and skeletal maturity constitute an important research topic nowadays.

### Current scenario of bone age assessment

Currently, the two most commonly used procedures for bone age assessment are the Greulich-Pyle (GP) and Tanner-Whitehouse (TW) methods [1]. Both are based on hand radiographs via the analysis of the epiphyses’ and diaphyses’ appearance. The GP method [7] is an atlas that contains reference images from hand radiographs collected from 1931 to 1942, from upper-middle class Caucasian children in Ohio, United States [1]. The attribution of bone age is done by comparing an individual’s hand radiograph with the reference images in the atlas [3]. The TW method [8] evaluates maturity scores for the radius, ulna, carpals and 13 hand short bones. Some of these bones are evaluated and categorized into stages ranging from A to I, then a total score is calculated, which is later converted into the bone’s age. The TW method was developed with data collected from 1950 to 1960, from children of average socioeconomic class in the United Kingdom. This method was further updated in 2001 based on new consistent patterns of development (secular trends) [3].

Besides their popularity, both GP and TW methods are criticized for many reasons. In a practical sense, since they are manually done by radiologists, the whole process can be time-consuming, which is aggravated by the increased demand for this activity due to the increase of individuals seeking refuge. Further, they can be prone to inter- and intra-rater variability, which raises ethical and legal issues, especially when considering that these assessments are done in relation to an individual being a minor or not [9].

In light of this scenario, a way to tackle these problems is to make use of automated methods, and a technology that is valuable in this matter is Machine Learning. This technology is already employed in many areas of medical research, ranging from genomics to diagnosis and prognosis of many disorders, aiming to find patterns in data and to provide useful estimates to improve decision making in the health area [10]. Machine Learning comprises a group of technologies that operates in the following way: firstly, the technique learns from a set of examples on how to perform a task, creating a model which encapsulates the knowledge to perform the task. Then, when new data is imputed, the model is able to correctly perform the learned task within an acceptable accuracy [11].

### Systematic review of the literature on bone age assessment

Given the motivations and scenario abovementioned, this paper contributes to the literature on age assessment as it describes a systematic literature review (SLR) that presents the state of the art on the use of Machine Learning techniques in the context of bone age and skeletal maturity assessment, a theme not previously addressed by SLRs. Hereby answering the research question: “How Machine Learning techniques are being employed in studies concerning youth age assessment (10 to 30 years)?”.

This paper is organized as follows: the Method section details the approach used to conduct the review, which followed the recommendations of the Preferred Reporting Items for Systematic Reviews and Meta-Analyses (PRISMA) Statement [12]; the Results section aggregates and synthetizes the data from the studies included in the review; the Discussion section argues about the results and provides considerations related to threats to validity; and finally the Conclusion section presents the final statements and comments on future work.

## Materials and methods

A systematic literature review (SLR) is an approach in which a significant and representative sample of the literature regarding a certain topic is identified, evaluated and interpreted. This is done by executing a comprehensive search following a pre-defined method that specifies the research questions the SLR aims to answer, the criteria used to include and exclude studies, how to assess their quality and how to extract and make the synthesis of the data [13–14]. The common motivations for executing an SLR are: to summarize next to all the evidence about a topic of interest; to find research gaps; to provide a grounding for new research; and to investigate how the research that is currently being done supports a certain hypothesis [13].

The SLR presented herein was conducted by four participants with different expertise (health, machine learning and health informatics). Throughout the text, references to the authors will use a notation, in which A1 refers to the first author; A2 refers to the second author, and so forth.

The main question this SLR aims to answer is: “How machine learning techniques are being employed in studies concerning youth age assessment (10 to 30 years)?”. This main question is further decomposed into the following five research questions.

- RQ1: Which machine learning techniques are being used in the age assessment studies?
- RQ2: What data characteristics (database’s origin, data collection mechanism, and ages) are being considered in the age assessment studies?
- RQ3: What type of medical imaging are being used in the studies?
- RQ4: What are the regions of interest being explored for the age assessment studies (hand, wrist, knee, etc) and what were the methods used to assess them?
- RQ5: What are the performances of the proposed methods?

For the RQ5, besides the SLR steps to summarize the information regarding the performances of the studies, a meta-analysis was also conducted. A meta-analysis is the application of statistical operations in order to synthetize the results of individual studies, so they can be understood in the context of all selected studies [15].

The protocol that guided this SLR can be found at: http://tiny.cc/4wuw8y.

### Search strategy

The search strategy used to find primary studies employed a search string built based upon the PICO framework, in which the main question is re-written in terms of four elements: Population, Intervention, Comparison and Outcome [14]. The “comparison” component was not used because the goal of SLR detailed herein was to characterize existing evidence. The components used for the automated searches are defined below.

- Population: Studies involving age assessment in youth.
- Intervention: Use of medical imaging.
- Outcome: Machine learning models for age assessment.

The resulting search string used to conduct the automated searches is shown in Table 1. The searches were performed in Pubmed, Scopus and Web of Science databases (with the necessary adaptations).

**Table 1:** Search String used in the Pubmed database.

Search Dates	21/03/2018 and 06/02/2019
(((“age assessment” OR “age appraisal” OR “age diagnostics” OR “age estimate” OR “age estimation” OR “age determination” OR “age prediction” OR “age testing”) AND (“bone age measurement” OR “bone age assessment” OR “bone maturity” OR “bone development” OR “bone age testing” OR “bone age tests” OR “skeletal maturity” “skeletal maturation” OR “bone examination” OR “skeletal development” OR “developmental assessment” OR “bone age” OR “skeletal age” OR “growth zone”)) AND (“Magnetic Resonance imaging” OR “MRI” OR “x ray” OR “x-ray” OR “xray” OR “Radiography” OR “computed tomography” OR “CT” OR “ultrasound” OR “ultrasonography” OR “medical imaging”) AND (“machine learning” OR “unsupervised Machine Learning” OR “supervised Machine Learning” OR”Classification” OR”Regression” OR”Kernel” OR”Support vector machines” OR”Gaussian process” OR”Neural networks” OR”Logical learning” OR”relational learning” OR”Inductive logic” OR”Statistical relational” OR”probabilistic graphical model” OR”Maximum likelihood” OR”Maximum entropy” OR”Maximum a posteriori” OR”Mixture model” OR”Latent variable model” OR”Bayesian network” OR”linear model” OR”Perceptron algorithm” OR”Factorization” OR”Factor analysis” OR”Principal component analysis” OR”Canonical correlation” OR”Latent Dirichlet allocation” OR”Rule learning” OR”Instance-based” OR”Markov” OR”Stochastic game” OR”Learning latent representation” OR”Deep belief network” OR”Bio-inspired approach” OR”Artificial life” OR”Evolvable hardware” OR”Genetic algorithm” OR”Genetic programming” OR”Evolutionary robotic” OR”Generative and developmental approaches”))	

### Study selection

Two sets of searches were executed in this SLR. The first was carried out on March 21^st^ 2018, and the second on February 6^th^ 2019, aiming to search for additional *evid*ence since the first search was conducted. The procedure for each search is detailed next.

After the removal of duplicates, the first search screened 148 studies, assessing titles and abstracts, guided by the inclusion and exclusion criteria (see Table 2). Four participants took part in this procedure, with A1 evaluating all of the papers and A2, A4 and A6 one third each. The Cohen’s Kappa was calculated as a measure of consensus between A1 and the other authors, resulting in strong agreements with all of them (0.73 with A4, 0.76 with A2 and 0.70 with A6). A total of 65 studies were selected to be fully read and assessed for eligibility.

**Table 2:** Inclusion and exclusion criteria for assessing the retrieved papers.

Inclusion Criteria	Exclusion Criteria:
• Be a primary study in English; AND • Have been published in the last 10 years; AND • Address the research on age assessment using medical imaging; AND • Be a study concerning the building of models for the purpose of age assessment using at least one machine learning technique; AND • Be a study in which the age assessment is analyzed through growth zones in joints; AND • Be a study regarding age assessment in living individuals.	• Be a secondary or tertiary study; OR • Have been published before 2007; OR • Be written on another language other than English; OR • Do not address research on age assessment using medical imaging; OR • Do not address the building of models for the purpose of age assessment; OR • Be a study concerning height prediction, or a specific syndrome/disease that affects normal growth; OR • Be a study in which the age assessment is not analyzed through growth zones in joints; OR • Be a study in which the study population is out of the range of 10 to 30 years old by 3 years or less; OR • Be a study in which the study population is post-mortem material.

One round of backward snowballing was performed on the 65 selected studies’ references, searching for additional evidence, and following an analogous process to the previous assessment. After screening 2124 items, 2 additional studies were selected. This step was performed by A1, and the remaining participants were consulted when necessary, based on their expertise.

In total, 67 studies were selected for eligibility assessment. These were fully read and re-assessed as per the criteria shown in Table 2. A quality assessment was also performed to minimize the chance of selecting studies with bias evidence. The quality questionnaire was adapted from Kitchenham’s guidelines [13] for performing SLRs, and is detailed in the SLR protocol. If a paper fell below the threshold of 8 points (out of 13) it would be removed from SLR due to quality reasons. The threshold was defined in meeting with all of the participants. A considerable amount of papers was removed from the SLR in this phase, because during the selection based on title and abstracts many abstracts did not contain all the information necessary to judge the inclusion and exclusion criteria, so they were included to be re-assessed when fully read. The responsible for fully reading the papers was A1, but A2, A4 and A6 were also consulted when necessary. A total of 22 studies were selected by the first search.

On the second search, 29 studies were screened analogously to the first search, resulting in 19 studies to be fully read and assessed for eligibility. The same quality assessment questionnaire was applied to the studies afterwards. A1 performed the selection process of the second search and A2, A4 and A6 were consulted when pertinent. In total, 4 studies were selected on the second search.

All of the selected studies were also assessed for the risk of cumulative evidence bias. This means that studies from the same group or same age assessment system were checked. Validation studies of a system that was already included in the set of selected studies were considered duplicates and not included in the final set, but will be further referenced when necessary. The final set of selected studies consisted of 26 papers.

### Data collection

Table 3 lists the data extracted from the studies. Besides these, other basic information was also extracted (i.e. authors, journal/source, year and type of publication).

**Table 3:** Data extracted from the selected studies.

Variables	Definition
Aim	The goal of the built model or the proposed study.
Age range of the subjects	The age range which the model for age assessment is concerned with.
Origin of the subjects	Characteristics related to the country/ethnicity of the subjects which the model is built upon.
Type of Image	Radiography, Magnetic Resonance Imaging (MRI), Ultrasound etc.
Regions of Interest for the images	Body part which the model analyses for the age assessment purposes.
Model Building Technique	The ML model building technique used to build an age assessment method.
Method used for age assessment	Method for age assessment that the model was built upon, if any (i.e. TW, GP).
Dataset size	The sample size utilized by the study.
Performance	Performance achieved by the best proposed model for bone age assessment.

### Data analysis

From the data collected, summary tables were built to summarize the results for the SLR and to answer the research questions. The meta-analysis of BAA performances was done through the average of the performances weighted by the sample sizes. The software used to perform the data analyses was Excel.

## Results

**Fig 1:**
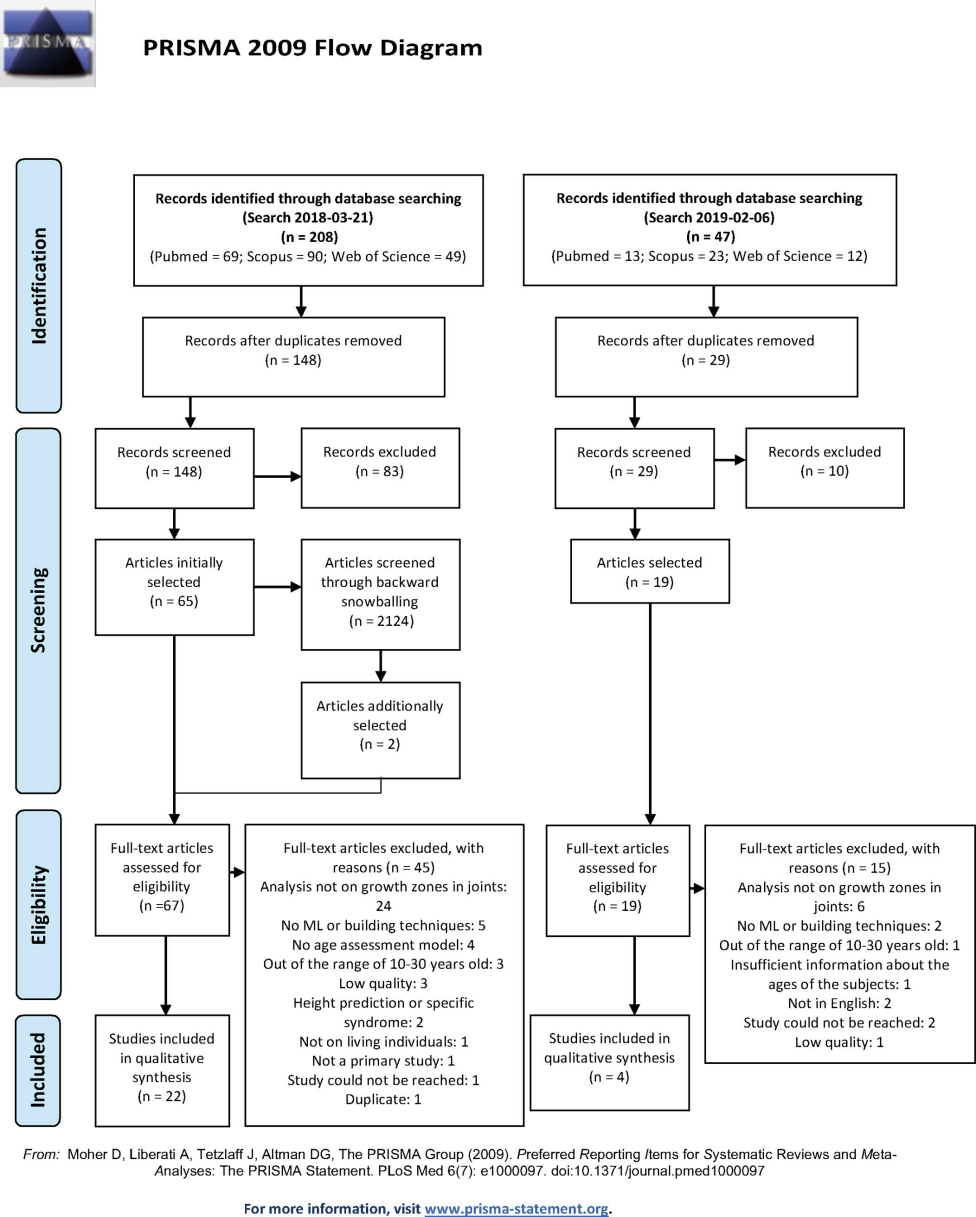
PRISMA flow chart.

The PRISMA flow chart that describes the process of selection of the articles that were included in this SLR is shown in Fig 1.

The study selection resulted in the assessment of eligibility of a total of 86 studies (67 from the first search and 19 from the second), from which 26 were included in the final set (22 from the first search and 4 from the second). The most common reasons for ineligibility were: the case where the age assessment method was not performed on growth zones in joints (e.g. dental assessment), totaling 30 studies; cases where no ML technique was employed, totaling 7 studies; cases where the study population was mostly out of the range of 10 to 30 years old; and cases where no age assessment model was proposed. The complete list of reasons for exclusions with its respective numbers is shown on Fig 1.

In the list of included papers, two studies were found to be from the same research group [16–17], and used the same data e related techniques. After being assessed for cumulative bias, it was decided that only the most recent [16] would be kept.

The complete list of included studies is presented in S1 Table, as supporting information (see S2 Table for the filled PRISMA checklist).

### Overview of the included Studies

Table 4 shows the summarized data arranged by the documented aims of the included studies. Eleven studies aimed to propose a new approach for an automatic Bone Age Assessment (BAA). Note that “automatic BAA system” means a fully automated approach without human intervention. These systems use a medical image as input and automatically detect the relevant region of interest (ROI), followed by the bone assessment age through an algorithm.

**Table 4:** Aims of the studies included in the SLR.

Aim	Number of Studies	Featured Studies
Proposed an automatic BAA system.	10	[16, 18–26]
Proposes a non-automatic BAA system.	7	[27–33]
Proposes a non-automatic BAA system. Also, test how reliable is the TW method in the western Australian population.	1	[34]
Proposes a non-automatic BAA system and compares to TW3	1	[35]
Proposed an automatic BAA system and compared with the manual BAA	1	[36]
Comparison between age assessment with MRI and Radiograph	1	[37]
Predicts the Skeletal Maturity Index (by Fisherman) from the chronologic age and compares the results of the American sample and Indonesian sample	1	[38]
Proposes a simplified version of the TW3 method	1	[39]
Examines sex-specific differences in the maturation time of the bones in the hand and wrist in two cohorts of children subjects to investigate secular trends.	1	[40]
Investigated the effect of the African-American ancestry, linear growth, body composition, and pubertal maturation on the skeletal maturation in a cohort of non-obese children and adolescents.	1	[41]
Investigates the persistence of the epiphyseal scar of the distal radius and its relationship with chronological age and sex.	1	[42]

Studies that aimed to propose a “non-automatic BAA system” have some degree of automation by the use of Machine Learning techniques, but are still dependent of some kind of human input. Human interventions in these systems are usually related to the manual description of the information contained in the medical image to be inputted in the system. The manual location of regions of interest (e.g. epiphyses and metaphyses) and the assessment of the image by radiologists as to specific stages of ossification or tissue analyses are examples of interventions performed by humans in these systems. The “non-automatic BAA systems” were present in 7 studies in the SLR.

Three studies, besides proposing a new BAA approach, compared its results to the traditional TW method, obtaining better results in terms of performance or time spent. The study by Fan et al. [37] proposed models to estimate age from the ossification stages of the knee using MRI and Radiograph images, yielding better results for the MRI images.

In addition to these, there were other studies that did not propose either automatic or non-automatic BAA, as follows: the study by Soegiharto et al. [38] compare the skeletal maturation index and the cervical vertebrae maturation in two cohorts, one composed of Indonesian subjects and other by Caucasian subjects. For both methods, the Indonesian children attained maturation stages from 0.5 to 1 year later in comparison with the Caucasian subjects. This study also proposed a model to predict the skeletal maturity index from chronological age that achieved good accuracy results in both cohorts. The study by Hsieh et al. [39] aimed to build on the TW method in an effort to simplify the RUS (Radius, Ulna and short bones) system assessment, in a way that only 9 out of the original 13 bones are assessed, reducing the time and effort needed for the BAA. An important study by Duren et al. [40] investigates the changes in bone maturation in two cohorts, one with subjects born between 1930 and 1964 and other with birth years between 1965 to 2001. The results showed that in the most recent cohort, the skeletal maturity was more advanced than in the earlier cohort for boys between ages of 0 to 8 years and 10 to 18 years, and girls between the ages of 4 to 17 years. McCormack et al. [41] conducted a longitudinal study with duration of 7 years, that performed annual assessments in children and adolescents from the African American and non-African American ancestry to investigate the effect of ancestry, height, BMI and pubertal maturation on the skeletal maturation. The results yielded that the subjects with African American ancestry had more advanced skeletal maturation, even when accounting for age, body composition and pubertal maturation. Lastly, the Davies et al. study [42] examine the presence of the epiphyseal scars in subjects after the bone growth ended. An epiphyseal scar is a thin layer of cartilage that remains between the diaphysis and epiphysis after they are completely fused. It is known that they remain for some time after the fusion, and the study in question investigates the bounds in which this occurs, concluding that subjects of the age of 50 still had visible epiphyseal scars.

### Employed machine learning methods

This section aims to answer the research question RQ1 “Which machine learning techniques are being used in the age assessment studies?”.

As shown in Table 5, the most frequently used techniques in the papers included in SLR were Regression-based methods (13 studies), followed by Artificial Neural Networks (8 studies) and Support Vector Machines (5 studies). Other less frequent techniques featured in the studies are: Bayesian Networks (2 studies), Decision Tress (1 study) and K-Nearest Neighbors (1 study). These results will be further detailed next, where we also provide a brief explanation of each of the techniques. Note that some studies used more than one technique.

**Table 5:** Summarized data about the ML techniques featured in the studies.

Machine Learning Techniques		Number of Studies
Regression-based methods	Linear regression [28–29, 29], Rule-based regression [28], General Linear Model [42], Mixed Effects Model [40–41], Logistic regression [40], Multiple Regression [38], Multivariate linear Stepwise regression [33], Polynomial Regression [34], Linear Discriminant analysis [31], Transition Analysis [30], Regression [18, 32, 37]	13
Artificial Neural Networks	Artificial Neural Networks [28, 35–36], Fuzzy Neural Networks [19],	4
Convolutional Neural Networks	Convolutional Neural Networks [22–25]	4
Support Vector Machines	Support Vector Machines [16, 21, 26, 28]	4
Bayesian Networks	Bayesian Networks [27], Gaussian process [28]	2
Decision Trees	Random Forest [20]	1
K-Nearest Neighbors	K-Nearest Neighbors [26]	1

The **Regression-based me**thods aim in finding the effect of a set of independent variables in a dependent variable of interest. This is done by identifying a non-deterministic function (prone to random errors) that models the influence of the independent variables towards the mean of the dependent variable [43]. Despite being simple, Regression methods require a pre-determined model for data fitting.

These were the techniques employed the most in the included studies, being typically applied in the studies proposing non-automatic BAA systems, that is, systems that will require some sort of human intervention (commonly the assessment of epiphyses or other aspect of the medical image). Such studies aimed for a degree of automation, but also investigated the influence of multiple Regions of Interest (ROI) in the BAA. The only exception was the study by Thodberg et al. [18], which describes the BoneXpert, an automatic BAA system, which locates the ROI in hand radiographs automatically, but computes the Bone Age itself using a series of regressions.

An **Artificial Neural Network** is a technique that performs multifactorial analyses. It consists of a multi-layer structure that contains nodes connected by weighted edges that establish an input layer, one or more hidden layers and an output layer. With known input and output values, the network is trained by adjusting the weights in an incremental way until the network’s output approaches the known output [44]. This technique is a powerful predictor that pairs well with image interpretation, but contrary to the regression case, this is a black box technique in which the results are not able to be interpreted in an intuitive way [45].

A specialization of the Artificial Neural Networks technique, which was frequently used in this SLR studies, is the **Convolutional Neural Network**. This technique enabled major advances in computer vision problems, where a computer needs to understand an input image to reconstruct its properties (i.e. color distributions, shapes, illumination etc) and perform a task such as localization, segmentation or detection of certain image elements [46]. One major difference from the traditional Artificial Neural Networks lays in its architecture, which is commonly composed by an input layer that receives an image as input, several convolutional and pooling layers, followed by several fully connected hidden layers, and finally the output layer [47]. The convolutional layers perform an image’s feature extraction, and are organized into feature maps; the pooling layers reduce the feature maps to a point of spatial invariance (not affected by distortions and translations of the input image); and the fully connected layers are responsible for the interpretation of the abstract feature representations learned by the previous layers [47]. In what concerns the training of such type of network, it is analogous to the traditional Artificial Neural Network; however, it requires greater computational power and larger amounts of data.

Six out of the 8 studies that featured Artificial Neural Networks and Convolutional Neural Networks aimed to propose automatic BAA systems, which take as input a medical image and return the bone age.

The **Support Vector Machines** technique was conceived to solve classification problems and it is one of the most recently proposed ML techniques. The classification process happens in the following way: given a set of labeled data (data points with known classes), the algorithm maps the data points into a feature space (that does not include the outcome variable), then it finds in this feature space the location that separates the classes in the with the maximum margin of separation [48]. This technique does not require a pre-defined model and works in scenarios where there is a high number of variables in comparison to the number of data points; however, it still requires some algorithm considerations to be made beforehand (i.e. the choice of a kernel function) [49].

In the studies included in this SLR, the support vector machines were employed mostly proposing automatic BAA systems, in three out of the four cases.

The **Bayesian Network** technique estimates the posterior probability of a data point being of a certain class, given a set of features. The learning process happens in two phases: first it learns the structure of the network, which is a direct acyclic graph composed of nodes (representing the features) and edges (representing the probabilistic dependencies), then it learns the conditional probability distribution of each node [50]. The advantages of Bayesian Networks are that they are able to encode the knowledge of domain experts in the graph structure and they can work with smaller amounts of data, in comparison to other ML techniques [51]. A great disadvantage is that Bayesian Networks may become impractical in problems with a large number of variables.

Both studies that employed this ML technique proposed non-automatic BAA systems; one of these studies (Cunha et. al [28]) compared various techniques, and the Bayesian Network was not the best performing one.

**Decision Trees** aim in building a tree structure to represent knowledge that can be easily translated into if-then rules. It follows a recursive learning process that begins with each feature being tested on how well it alone can classify the data points into a certain class. The best one is set as the root node and the descendant nodes are set as the possible values (or ratios) of the selected feature. Then, the data points are sorted according to this rule. This process repeats until there are no possible splits [11]. The advantages of the Decision trees are that they are easy to interpret, to understand the interactions between features, and they do not depend on a pre-defined model [52]. The disadvantages are that they can be unstable and prone to overfitting [53].

The Decision Trees technique was employed in only one study by Urschler et al. [20], but in its ensemble form: Random Forest, which aims to address the abovementioned disadvantages of decision trees. That study proposed as a non-automatic BAA system.

The **K-Nearest Neighbors (KNN)** algorithm maps a data point of unknown class to a feature space. Then it assigns it to the class pertaining its k nearest neighbors. In this case, the concept of near is usually related to the Euclidean distance, but other measures can apply [54]. This technique is very simple and easy to implement, but it also considers all the features as equal in terms of importance, what can be a problem if superfluous features are inserted in the feature space [55].

The K-Nearest Neighbors technique was employed in the study by Harmsen et al. [26], which proposed a non-automatic BAA system. This study compared the KNN to SVM, and KNN presented worse performance.

### Sample data characteristics

This section presents the results regarding the research question RQ2 “What data characteristics (database’s origin, data collection mechanism, and ages) are being considered in the age assessment studies?”

Table 6 shows the summary regarding the data’s origin in the samples of the included studies, grouped by region. Most of the studies utilized subjects from diverse countries of west Europe (8 studies), followed by North America (7 studies), Asia (6 studies) and finally Australia (2 studies). Two studies made use of samples from two regions, one used to build a model, and the other to validate the same model. Also, one of the studies reported no data origin, but mentioned the ethnicity of the sample to be Caucasian.

**Table 6:** Summarized data regarding the origin of the subjects in the featured studies.

Region	Data’s Origin	Featured Studies	Number of Studies	
**Europe**	Italy	[31–32]	2	**8**
	Austria	[20]	1	
	Denmark	[18]	1	
	Ireland	[29]	1	
	IRMA Database	[26]	1	
	Portugal	[28]	1	
	Scotland	[42]	1	
**North America**	United States of America	[22, 40–41]	3	**7**
	Digital Hand Atlas of the USC	[16, 21]	2	
	RSNA Pediatric Bone Age Challenge 2017 Database	[23–24]	2	
**Asia**	China	[33, 35–37]	4	**6**
	Taiwan	[19, 39]	2	
**Australia**	Australia	[30, 34]	2	**2**
**Mixed**	RSNA Pediatric Bone Age Challenge 2017 Database and China	[25]	1	**2**
	Unite States of America and Indonesia	[38]	1	
**Non-Specified**	Caucasian	[27]	1	**1**

Regarding the studies’ data collection, 20 out of 26 made use of private databases of medical images, or queries in hospital databases, but six studies made use of public databases for their experiments. These were:

- Digital Hand Atlas of the University of Southern California (USC), which contains 1097 radiograph left hand images of subjects ranging from 0+ to 18 years of age, of both male and female genders and of different ethnicities, denoted as: African American, Asian, Caucasian and Hispanic.
- Radiological Society of North America (RSNA) Pediatric Bone Age Challenge 2017 Database, which was an initiative that brought 37 teams to develop algorithms for BAA. This database was developed by Stanford University and consisted of 12611 hand radiographs from male and female subjects, ranging from 0+ to 19 years of age. This database was only publicly available during the Challenge 2017 period.
- Image retrieval in Medical Applications (IRMA), which is a radiologic database brought together by the Aachen University of Technology in Germany, where one of its goals is to enable research in diverse automated classification problems involving radiographs. It comprises images from many body regions.

Note that few studies reported the ways used to verify the origin or ethnicity of the subjects in the sample; several reported that this information was not available, which may be the case when using private datasets. This problem is less significant in the case of the Asian studies, which employed data on subjects from China and Taiwan, since the Han ethnicity is very prominent in these countries reaching more than 90% of the total population and being the largest ethnical group in the world [56–57]. Other factors such as socioeconomic aspects were also not explored in the studies.

Regarding the subjects’ age ranges for the samples used in studies detailed in this SLR, these were divided into 4 categories:

- Comprehensive Sample: sample contains data on subjects younger than 18 up to older than 18
- Bordering the age of 18: The maximum age of the sample is exactly 18 years’ old
- Younger Subjects: the entire sample contains data on subjects younger than 18
- Older Subjects: the entire sample contains data on subjects older than 18

Table 7 shows that most of the studies, 17 out of 26, are employing a ‘comprehensive’ or a ‘bordering 18 years old’ sample, what may suggest that BAA is focused in assessing this specific age. In fact, all of the North American (7 studies) and half of the European studies (4 studies) focused their research on these types of sample. Eight studies were concerned with age assessment of younger subjects. There is only one case of the category Older Subjects, which is the study by Davies et al. [42], which aims to investigate the persistence of epiphyseal scars after the fusion of the diaphysis and epiphysis. This study warns about methods that consider the complete obliteration of the epiphyseal scars, since there are cases in which they remain not entirely fused until the fifth decade of life.

**Table 7:** Summarized data regarding the age ranges of the subjects in the features studies.

Sample’s Characteristics	Featured Studies	Number of Studies
Comprehensive Sample	[20, 23–24, 27–30, 34, 36–37, 40]	11
Younger Subjects	[18–19, 31–33, 35, 38–39]	8
Bordering the age of 18	[16, 21–22, 25–26, 41]	6
Older Subjects	[42]	1

### Age assessment methods

Table 8 summarizes the data regarding the BAA methods, which is the focus of RQ3: “What type of medical imaging are being used in the studies?”. Evidence shows that to date the research being conducted in BAA favors the use of Radiographs over other types of medical imaging, with 22 out of 27 studies utilizing this method. Two other studies used radiographs in conjunction with MRI. The study by Hillewig et al. [27] made use of radiographs for the regions of hand and wrist, and MRI for the clavicle. Fan et al. [37] utilized these two methods in the knee region, but comparing the performance of both for the BAA, which yielded better results for the MRI. The studies that only featured MRI as the method of choice [20, 35] investigated the hand and wrist regions and features related to the appearance of osseous structures in terms of signal intensity provided by the MRI. The only case where a computer tomography (CT) assessed the Bone Age in the region of the clavicle was the study by Franklin and Flavel [30].

**Table 8:** Methods used for BAA.

Type of Image	Number	Featured Studies
Radiograph	21	[16, 18–19, 21–26, 28–29, 31–34, 36, 38, 39–42]
MRI	2	[20, 35]
MRI, Radiograph	2	[27, 37]
CT	1	[30]

### Regions of interest

This section presents the summarized data used to answer RQ4: “What are the regions of interest being explored for the age assessment studies (hand, wrist, knee, etc) and what were the methods used to assess them?”.

As shown in Table 9, the research is very much focused on the hand region for estimating Bone Age, accounting to 22 out of the 27 studies in this SLR; however, note that only in seven cases the hand alone was employed in the models. Next, the wrist was the second mostly investigated region, present in 17 out the 27 studies; however, in the same way as the wrist, it was employed exclusively in only two studies. In most of cases, data on both hand and wrist are used, what can be explained by the easiness of getting both regions in the same image. Other less frequent ROIs present in the BAA studies are the knee (2 studies) and clavicle (2 studies). It is important to notice that in the case of the teeth and cervical, even if they are ROI, this SLR focused in the BAA analyzed through growth zones in joints, so they were classified as “Other variables”.

**Table 9:** Summarized data regarding ROI, types of medical image, additional variables and techniques for BAA in the studies of the SLR.

Regions of Interest	Type of Image	Other Variables	Techniques for BAA	Featured Studies
**Hand, Wrist**	MRI	None	Computer Vision	[20]
	Radiograph	None	Computer Vision	[22, 24–25]
	Radiograph	None	GP, TW, Computer Vision	[18]
	Radiograph	None	TW	[34, 36, 39]
	Radiograph	None	Fels Method [58]	[40]
	Radiograph	Cervical Assessment	Skeleton Maturation Index by Fishman [59]	[38]
	Radiograph	Dental Assessment	GP, TW	[31]
	Radiograph	DNA Methylation, Dental Assessment	GP, TW, DNA Methylation	[33]
	Radiograph	BMI, Height, Tanner scale, Fat Mass, Lean Mass	GP	[41]
	MRI	Weight, Height	TW	[35]
**Hand**	Radiograph	None	Computer Vision	[16, 23, 26]
	Radiograph	None	Gilsanz and Ratib [60]	[21]
	Radiograph	None	Own Method	[19]
	Radiograph	None	TW	[28]
**Wrist**	Radiograph	None	Cameriere et al. [61]	[32]
			Own method	[42]
**Knee**	Radiograph	None	O’Connor et al. [62]	[29]
	Radiograph and MRI	None	Kramer et al. [63]	[37]
**Clavicle**	CT	None	Schmeling et al. [64]	[30]
**Hand, Wrist, Clavicle**	Radiograph and MRI	None	Schmeling et al. [64], Kreitner et al. [65], GP	[27]

Other aspect that can be evidenced is that only 12 out of the 26 studies explored only one ROI. Furthermore, only five studies employed additional variables not related to the assessment of growth zones: dental assessment, cervical assessment, weight, height, DNA Methylation, Tanner scale, fat mass, lean mass and BMI.

With regard to BAA techniques, the research deviated from the classic BAA of growth zones—the TW and GP methods, which were only present in five studies each. Furthermore, computer vision techniques, which do not depend on any pre-existent guide, still have a subtle presence in the BAA research, accounting for 8 out of 26 studies. This is enabled by the advances in computing power and the existence of a large amount of digital labeled data [45].

Other techniques for BAA that were employed in specific ROIs are summarized in the Table 10. Following the same trend as TW and GP, these are either based on maturity scores or atlases.

**Table 10:** Techniques for BAA by ROI.

ROI	Techniques for BAA
**Hand, Wrist**	**Fels Method:** This method was proposed as a less subjective way to assess skeletal age. It considers ossification, radiopaque densities, bony projection, shape changes and fusion. It comprises 98 indicators of bone maturity, where 85 are categorical and are 13 continuous (epiphyseal and metaphyseal fusion ratios) [58].
	**Skeleton Maturation Indicators by Fishman:** It assesses the skeletal maturity based on the following indicators: width of the epiphysis compared to the diaphysis (third and fifth fingers), gapping of epiphysis (third and fifth fingers), fusion of epiphysis and diaphysis (third, fifth fingers and radius) and ossification of adductor sesamoid of the thumb [59].
**Hand**	**Gilsanz and Ratib:** This method consists of a digital hand atlas with reference images of 29 classes from the ages of 0 to 18 with an additional class to represent subjects older than 18. [60]
**Wrist**	**Cameriere et al.**: This is a quantitative method that proposes a mathematical formula based on the ratio of the carpal area and the total area of carpal bones and epiphyses of the radius and ulna [61].
**Knee**	**O’Connor et al.**: A method that proposes five stages of epiphyseal fusion for the femur, tibia and fibula bones. It uses the frontal and lateral radiograph image of the knee [62].
	**Krammer et al.:** A method that proposes five stages of epiphyseal fusion (from 1 to 5 of which stages 2 and 3 have 3 sub stages each) of the distal femur [63].
**Clavicle**	**Schmeling et al.:** This method defines 5 stages of ossification of the medial clavicular epiphysis [64].
	**Kreitner et al.:** This method is similar to the one proposed by Schmeling et al. (63), but instead of 5 stages there are 4, and the fourth stage is comparable to a combination of the stages 4 and 5 from the Schmeling classification [65].

### Performance of the techniques in the included studies

The information regarding the performance of the techniques employed in the studies that proposed systems for BAA was extracted and it is summarized in this section. Results show that a wide variety of different metrics were used to measure a technique’s performance what makes comparisons between studies difficult. The most commonly used metric was the mean average error (MAE), which is the average of all the absolute errors, used by seven studies. Table 11 shows the studies that used this metric, and their performance information. The reported performances were aggregated by means of the average of the performances weighted by the sample sizes to perform the meta-analysis, so to understand the general performance of BAA systems.

**Table 11:** Performance of the comparable studies in terms of the mean absolute error (MAE) in months.

Proposed Method	Dataset size	Age Range	Performance in MAE (months)	Commentary
Ren et al. (2018) [25]	12480	0–18	5.2	2017 RSNA Pediatric Bone Age challenge entry, but the method is tested in a different sample of age range 0–18.
Kashif et al. (2016) [21]	1101	0–18	7.26	
Iglovikov et al. (2018) [24]	11600	0–19	7.52	2017 RSNA Pediatric Bone Age challenge entry
Zhao et al. (2018) [23]	12611	0–19	7.66	2017 RSNA Pediatric Bone Age challenge entry
Harmsen et al. (2013) [26]	1097	0–19	9.96	
Urschler et al. (2015) [20]	102	13–20	10.2	
Cunha et al. (2014) [28]	887	7–19	10.16	

The result of the weighted average was 9.96 MAE (months), which could indicate that BAA systems could reliably predict the bone age of a subject from zero to 19 years old, but this results should be regarded with caution since this statistic was based on only 7 out of the 20 studies that proposed BAA systems. The studies that employed the MAE metric had relatively similar age ranges, but still varied in two of the studies which did not consider infant and children subjects. Also, not all of the studies had a uniform sample in terms of age distribution. In the RSNA challenge database, although the sample was large enough, only 0.1% of the sample was composed of subjects of 18 years old and older, which is a very important bone age to be predicted for legal reasons.

The remaining studies that proposed BAA systems, besides using a wide variety of different performance metrics, also were more heterogeneous in terms of age ranges, making the comparison between them not viable. The results for the performances of the employed techniques by the studies are shown in Table 12.

**Table 12:** Performance of the non-comparable studies.

Proposed Method	Dataset size	Age Range	Performance	Commentary
Shi et al. (2017) [33]	124	6–15	0.47 (male) and 0.33 (female) MAE (years)	
Haak et al. (2013) [16]	1097	0–18	0.73 RMS	The RMS metric is the root mean squared error is the square root of the mean square error
Thodberg et al. (2009) [18]	1559	2–17	0.42 (GP), 0.80 (TW) MSE	The MSE is the mean squared error and measures the average squared differences between the estimated and observed values.
Lin et al. (2012) [19]	600	0–14	0.26 MSE	Predicts a bone age cluster instead of bone age.
Lee et al. (2017) [22]	8325	5–18	61.40% (male) and 57.32% (female) accuracy	
Maggio et al. (2016) [34]	360	0–24	1.31 (male) and 2.37 (female) SEE (years)	SEE is the standard error of estimate and measures the variation from the regression line.
De Luca et al. (2016) [32]	332	1–16	0.38 median of the absolute values of residuals	
O'Connor et al. (2014) [29]	221	9–19	-2.0 to +2.9 (male) and -2.3 to +2.4 (female) range residuals	
Pinchi et al. (2016) [31]	274	6–17	80.4% (male) and 83.3% (female) accuracy	The TW method performed better than the proposed method for male subjects (negative results).
Tang et al. (2018) [35]	79	12–17	0.13 (male) and 0.08 (female) mean disparity (years)	The mean disparity is a metric that compares the mean chronological age of all subjects to the mean estimated age for all subjects.
Hsieh et al. (2011) [39]	534	2–15	96.2% (male) and 95% (female) relative accuracy	Measures the relative accuracy between the proposed method and the TW method.
Franklin, D.; Flavel, A. (2015) [30]	388	10–35	NA	Create stages of ossification for the clavicle and compares to the bone age.
Hillewig et al. (2013) [27]	220	16–26	NA	Calculates the probability of being of bone age younger or older than 18 instead of the actual bone age.

## Discussion

### Discussion of the current evidence

This SLR’s main findings can be summarized as follows: (i) Most studies aimed to propose an automatic BAA system; (ii) The BAA research has focused on hand and wrist radiographs; (iii) Most studies made use of samples from either the United the States or from West Europe; (iv) Studies that considered ethnical differences were scarce and socioeconomic aspects were inexistent; (v) The estimations on Bone Age were using samples where subjects’ age ranged from below to above 18 years of age, or bordering 18 years of age; (vi) The average performance weighted by sample size of the compared studies resulted in a MAE of 9.96 months, but there is still high heterogeneity in the studies what makes the comparing them a challenge.

The evidence gathered in this SLR suggests a clear trend towards automating the age identification within the context of BAA research. Most studies aimed at proposing automatic systems that would not require human intervention; however, a considerable amount of other studies proposed systems that do not automate age identification but reduce the dependability upon human input. We believe that either solution can be motivated by the following issues: i) to reduce the subjectivity of the traditional BAA methods, which depend upon radiologists’ judgment and experience, and, as a consequence, can lead to inter-rater and intra-rater variability [66–67]; ii) the traditional BAA methods are a time consuming activity, which demand is increasing [66]; and iii) more automatic solutions could reduce assessment costs as they would require radiologists to spend less time in such this activity [9].

Note that in relation to using an automated solution to chronological age identification, the use of ML technologies appears to be a significant enabler of automatic BAA solutions; this is observed in particular when using Convolutional Neural Networks for computer vision tasks, which contrast with the use of regression-based methods techniques by systems that still require some human intervention. As a remark, the GP atlas, which is being used nowadays as a BAA, was not created to determine chronological age, but to compare the skeletal development of children and adolescents to their chronological age [5]; nevertheless some studies still used this method as the base for their investigations on age assessment.

Further, this SLR also showed that the research on BAA systems has focused upon methods that employ hand and wrist radiographs. However, such choice of medical imaging on healthy children and adolescents—the use of radiographs, hence radiation, without therapeutic purposes could raise ethical issues [5]. When comparing radiographs with other forms of medical imaging, such as Magnetic Resonance Imaging (MRI), there is the argument that the latter is more expensive; however, on the other hand, the MRI technology offers better contrast resolution, which in turn offers a more accurate analysis of the growth plate, especially considering 3.0-T MRI [68]. The small presence of the MRI technology on the research on BAA could be considered a gap in the research.

With regard to the predominant use in this SLR studies of hand and wrist as the focused ROI, one can argue that this is a small area that contains a large concentration of epiphyseal plates, thus making it easier to gather images from this region without much effort. Also, very few studies investigated the BAA with more than one ROI whenever they were not using hand and wrist, suggesting a gap in the current research.

This SLR also evidenced that two regions seem to show predominant interest in such research—the United States and West Europe; such attention resulted in the creation of databases of medical imaging (USC, IRMA and RSNA), and the Pediatric Bone Age Challenge 2017, which can be viewed as an effort to have standardization as basis of comparison of different studies on this topic.

In what concerns a sample’s origin, most studies did not report on mechanisms used to document it. In addition, the ethnicity aspect was not much explored in the studies and the socioeconomic element was not investigated, what can be viewed as a gap in the current research on BAA. Only two studies (Soegiharto et al. [38] and McCormack et al. [41]) approached the effects of ethnicity in skeletal maturation. Besides the contradictory evidence in the literature about its influence [5], in this SLR both studies reported on differences in skeletal maturation on Indonesians and African Americans, in comparison to Caucasians, in the way that the first mature later and the second matures earlier. In contrast to that, the study by Thodberg et al. [18], which proposed a BAA system that is currently in commercial use—the BoneXpert, investigated the BAA issue using samples of Japanese [69], Dutch [70] and American subjects of four ethnicities (African American, Asian, Caucasian and Hispanic) [71].

Furthermore, regarding the subjects’ age in the samples used by this SLR’s included studies, most age ranges either included or bordered the age of 18 years. The interest for this particular age is justified by the legal systems in many countries, where younger than 18 individuals are classified as minors.

In regards to the meta-analysis of performances, it was evidenced a high heterogeneity in terms of age-ranges, dataset sizes and performance metrics that make the comparison between studies a challenge. The seven comparable studies had quite similar age-ranges and resulted in a weighted average of 9.96 MAE (months), but caution should be made as the age distribution was not uniform in all of the studies.

### Limitations

With regard to this SLR’s limitations, they include whether a suitable large representative sample of relevant included studies were selected, and also the non-medical expertise of A1. The first issue was mitigated via using a more inclusive selection strategy, i.e., whenever a paper’s abstract did not present all the information needed for its inclusion or exclusion, it would be included in the first phase to be fully read later. As for the second issue, A1’s lack of medical expertise of A1 was mitigated by consulting A3 whenever necessary.

### Future perspectives

The results of this SLR presented trends and gaps in the current research on age assessment that should be addressed, such as other common factors that could influence delay or acceleration of skeletal maturity and the further investigation of other ROIs for BAA.

## Conclusion

This paper detailed an SLR on the topic of age assessment in growth zones research with the use of ML techniques, which resulted in the selection of a final set of 27 studies. These studies were summarized in terms of ML techniques applied, sample data, age assessment methods and regions of interest.

Our findings indicate the focus of the research on investigating the hand and wrist ROIs with radiographs, with most of the samples from the United States or West Europe. It has also pointed out gaps in the research, such as few studies on different ethnicities, no studies considering socioeconomic differences, and few studies considering more ROIs other than hand and wrist.

## Supporting information

S1 Table Included studies in the systematic literature review. (PDF)

S2 Table PRISMA checklist. (PDF)
