# Supplementary material for: Bone age assessment with various machine learning techniques: A systematic literature review and meta-analysis
Source: PLoS One. 2019 Jul 25;14(7):e0220242. doi: 10.1371/journal.pone.0220242 (PMC6657881; doi:10.1371/journal.pone.0220242)
Supplement: S1 Table — (PDF) [file pone.0220242.s001.pdf]

S1 Table. Included studies in the systematic literature review

	Title	Author	Publication Title	Publication Year	Automatic or Non-Automatic BAA	ROI	Type of image
1	A new formula for assessing skeletal age in growing infants and children by measuring carpals and epiphyses of radio and ulna [31]	De Luca, S. et al.	Journal of Forensic and Legal Medicine	2016	Non-Automatic	Wrist	Radiograph
2	Accurate Age Determination for Adolescents Using Magnetic Resonance Imaging of the Hand and Wrist with an Artificial Neural Network-Based Approach [34]	Tang, F. H. et al.	Journal of Digital Imaging	2018	Non-Automatic	Hand, wrist	MRI
3	Age prediction formulae from radiographic assessment of skeletal maturation at the knee in an Irish population [28]	O'Connor, J. E. et al.	Forensic Science International	2014	Non-Automatic	Knee	Radiograph
4	Automatic bone age assessment based on intelligent algorithms and comparison with TW3 method [35]	Liu, J. et al.	Computerized Medical Imaging and Graphics: The Official Journal of the Computerized Medical Imaging Society	2008	Automatic	Hand, wrist	Radiograph
5	Bone age assessment using support vector regression with smart class mapping [15]	Haak, D. et al.	Proceedings of SPIE - The International Society for Optical Engineering	2013	Automatic	Hand	Radiograph
6	Bone age cluster assessment and feature clustering analysis based on phalangeal image rough segmentation [18]	Lin, H. H. et al.	Pattern Recognition	2012	Automatic	Hand	Radiograph
7	Combining dental and skeletal evidence in age classification: Pilot study in a sample of Italian sub-adults [30]	Pinchi, V. et al.	Legal Medicine (Tokyo, Japan)	2016	Non-Automatic	Hand, wrist	Radiograph
8	CT evaluation of timing for ossification of the medial clavicular epiphysis in a contemporary Western Australian population [29]	Franklin, D.; Flavel, A.	International Journal of Legal Medicine	2015	Non-Automatic	Clavicle	Computer Tomography

9	DNA methylation markers in combination with skeletal and dental ages to improve age estimation in children [32]	Shi, L. et al.	Forensic Science International. Genetics	2017	Non-Automatic	Hand, wrist	Radiograph
10	Do Secular Trends in Skeletal Maturity Occur Equally in Both Sexes? [39]	Duren, D. L. et al.	Clinical Orthopaedics and Related Research	2015	Non-Automatic	Hand, wrist	Radiograph
11	Feature description with SIFT, SURF, BRIEF, BRISK, or FREAK? A general question answered for bone age assessment [20]	Kashif, M. et al.	Computers in Biology and Medicine	2016	Automatic	Hand	Radiograph
12	Forensic age estimation of living persons from the knee: Comparison of MRI with radiographs [36]	Fan, F. et al.	Forensic Science International	2016	Non-Automatic	Knee	Radiograph, MRI
13	Fully Automated Deep Learning System for Bone Age Assessment [21]	Lee, H. et al.	Journal of Digital Imaging	2017	Automatic	Hand, wrist	Radiograph
14	Impact of ensemble learning in the assessment of skeletal maturity [27]	Cunha, P. et al.	Journal of Medical Systems	2014	Non-Automatic	Hand	Radiograph
15	Magnetic resonance imaging of the sternal extremity of the clavicle in forensic age estimation: towards more sound age estimates [26]	Hillewig, E. et al.	International Journal of Legal Medicine	2013	Non-Automatic	Hand, wrist, clavicule	MRI (clavicle), Radiograph (hand, wrist)
16	Paediatric bone age assessment using deep convolutional neural networks [23]	Iglovikov, V.I. et al.	Lecture Notes in Computer Science	2018	Automatic	Hand, wrist	Radiograph
17	Regression Convolutional Neural Network for Automated Pediatric Bone Age Assessment from Hand Radiograph [24]	Ren, X. et al.	IEEE journal of biomedical and health informatics	2018	Automatic	Hand, wrist	Radiograph
18	Relative Skeletal Maturation and Population Ancestry in Nonobese Children and Adolescents [40]	McCormack, S.E. et al.	Journal of Bone and Mineral Research	2017	Non-Automatic	Hand, wrist	Radiograph
19	Simplified radius, ulna, and short bone-age assessment procedure using grouped-Tanner-Whitehouse method [38]	Hsieh, C. et al.	Pediatrics International: Official Journal of the Japan Pediatric Society	2011	Non-Automatic	Hand, wrist	Radiograph
20	Skeletal age estimation in a contemporary Western Australian population using the Tanner-Whitehouse method [33]	Maggio, A. et al.	Forensic Science International	2016	Non-Automatic	Hand, wrist	Radiograph

21	Skeletal maturation in Indonesian and white children assessed with hand-wrist and cervical vertebrae methods [37]	Soegiharto, B.M. et al.	American Journal of Orthodontics and Dentofacial Orthopedics	2008	Non-Automatic	Hand, wrist	Radiograph
22	Support vector machine classification based on correlation prototypes applied to bone age assessment [25]	Harmsen, M. et al.	IEEE journal of biomedical and health informatics	2013	Automatic	Hand	Radiograph
23	The BoneXpert method for automated determination of skeletal maturity [17]	Thodberg, H.H. et al.	IEEE transactions on medical imaging	2009	Automatic	Hand, wrist	Radiograph
24	The persistence of epiphyseal scars in the distal radius in adult individuals [41]	Davies, C. et al.	International Journal of Legal Medicine	2016	Non-Automatic	Wrist	Radiograph
25	Versatile Framework for Medical Image Processing and Analysis with Application to Automatic Bone Age Assessment [22]	Zhao, C. et al.	Journal of Electrical and Computer Engineering	2018	Automatic	Hand	Radiograph
26	What automated age estimation of hand and wrist MRI data tells us about skeletal maturation in male adolescents [19]	Urschler, M. et al.	Annals of Human Biology	2015	Automatic	Hand, wrist	MRI

BAA, Bone Age Assessment; ROI, Region of Interest; MRI, Magnetic Resonance Imaging.
